# Changes of PK/PD of Meropenem in patients with abdominal septic shock and exploration of clinical rational administration plan: a prospective exploratory study

**DOI:** 10.1038/s41598-024-60909-7

**Published:** 2024-05-03

**Authors:** Youquan Wang, Hongxiang Li, Dongxia Wang, Yuting Li, Yangyang Shen, Yao Fu, Yanhua Li, Meng Gao, Dong Zhang

**Affiliations:** 1 Department of Critical Care Medicine, The First Hospital of Jilin University, Chaoyang District, Changchun City, 130021 Jilin Province China; 2 Yantai Yeda Hospital, Shandong, China

**Keywords:** Meropenem, Pharmacokinetics/pharmacodynamics, Septic shock, Monte Carlo simulation, Intensive care, Diseases, Health care

## Abstract

This study aimed to explore the changes of pharmacokinetic parameters after meropenem in patients with abdominal septic shock after gastrointestinal perforation, and to simulate the probability of different dosing regimens achieving different pharmacodynamic goals. The study included 12 patients, and utilized high performance liquid chromatography-tandem mass spectrometry to monitor the plasma concentration of meropenem. The probability of target attainment (PTA) for different minimum inhibitory concentration (MIC) values and %fT > 4MIC was compared among simulated dosing regimens. The results showed that in 96 blood samples from 12 patients, the clearance (CL) of meropenem in the normal and abnormal creatinine clearance subgroups were 7.7 ± 1.8 and 4.4 ± 1.1 L/h, respectively, and the apparent volume of distribution (Vd) was 22.6 ± 5.1 and 17.2 ± 5.8 L, respectively. 2. Regardless of the subgroup, 0.5 g/q6h infusion over 6 h regimen achieved a PTA > 90% when MIC ≤ 0.5 mg/L. 1.0 g/q6h infusion regimen compared with other regimen, in most cases, the probability of making PTA > 90% is higher. For patients at low MIC, 0.5 g/q6h infusion over 6 h may be preferable. For patients at high MIC, a dose regimen of 1.0 g/q6 h infusion over 6 h may be preferable. Further research is needed to confirm this exploratory result.

## Introduction

Sepsis is a life-threatening organ dysfunction caused by an altered host response to infection^1^, and it is one of the main causes of admission to the intensive care unit (ICU) and one of the most common complications in the ICU^2^. Despite increasing awareness of sepsis and medical technology in recent years, there are still more than 30 million sepsis patients worldwide each year, and the latest data indicate that the mortality rate of sepsis patients is as high as 26.7%^3^. Abdominal sepsis is one of the leading causes of mortality among patients in the ICU, with a reported overall mortality rate of 10.5% for complex intra-abdominal infections (IAIs) worldwide. In particular, the mortality rate for septic shock patients with complicated abdominal sepsis can be as high as 36.5%^4^. Early, prompt, and efficacious antimicrobial intervention holds the potential to diminish mortality rates and enhance patient outcomes among this patient cohort^5–7^. However, the escalating utilization of broad-spectrum antibiotics has resulted in a surge of resistance among Carbapenem-resistant organisms and other bacteria. Subsequently, this has posed substantial challenges to antimicrobial therapy in effectively combating infections^8–10^. Hence, the paramount concern in the present medical landscape lies in the judicious and effective administration of antibiotics while simultaneously striking a delicate equilibrium between therapeutic efficacy and the imperative task of preventing and treating CROs. This critical balance assumes utmost significance in the management of sepsis.

Meropenem, a β-lactam antibiotic and a member of the carbapenem class, is effective against Gram-negative rods, Gram-positive cocci, and anaerobic bacteria. It is commonly used for empirical therapy in patients with sepsis or septic shock in the ICU^11^. As a time-dependent antibiotic, the best pharmacokinetic/pharmacodynamic (PK/PD) parameter reflecting efficacy is the percentage of time that the free drug concentration remains above the minimum inhibitory concentration (%fT > MIC). The traditional target value for carbapenems is achieving 40%fT > MIC for antibacterial effect, and animal experiments have shown that maximum effect is reached when the coverage of dosing intervals reaches 60–70%^12,13^. However, some studies suggest that a 100%fT > MIC target is more suitable for critically ill septic patients, as it can improve clinical efficacy and reduce the development of antibiotic resistance^14^. Meropenem exhibits maximal bactericidal activity at about 4 times the MIC, but increasing concentrations from 5 to 20 times the MIC does not enhance its bactericidal effect and may even result in toxicity at concentrations of 8 times the MIC^15^. Therefore, recent research recommends setting the treatment target at 100%fT > 4MIC^16^. However, the recommended dosages for treating sepsis are based on PK data obtained from patients with mild sepsis, and the current PK parameters of meropenem in patients with abdominal sepsis are still unknown^17,18^. CIAOW study shows that among the many causes of abdominal infection, gastrointestinal perforation is a risk factor for in-hospital mortality^4^. Therefore, we explored the PK parameters of meropenem in patients with abdominal septic shock following digestive tract perforation, and evaluated the probability of target attainment (PTA) of meropenem under different dosing regimens in this patient population. This study provides a theoretical basis for the rational application of meropenem in patients with abdominal sepsis in the ICU.

## Methods

### Protocol and registration

We conducted a prospective exploratory study on patients with abdominal septic shock after gastrointestinal perforation surgery who were admitted to the ICU of the First Hospital of Jilin University from January to June 2023. Inclusion criteria were as follows: (1) age ≥ 18 years; (2) patients with abdominal septic shock caused by gastrointestinal perforation (diagnostic criteria for septic shock according to Sepsis 3.0 released by the Society of Critical Care Medicine and the European Society of Intensive Care Medicine^19^). Exclusion criteria were as follows: (1) pregnant or lactating patients; (2) patients with acute kidney injury (AKI) or chronic kidney disease; (3) patients using sulbactam, glycerol phenylbutyrate, or sodium phenylbutyrate; (4) patients allergic to this drug or other carbapenem antibiotics; (5) patients who received meropenem within 7 days before ICU admission; (6) patients who did not sign an informed consent form; (7) patients participating in other clinical studies. Patient withdrawal: (1) patients who did not follow the experimental protocol; (2) patients who voluntarily or involuntarily withdrew from the study due to various reasons.

This study was approved by the Chinese Clinical Trials Registry (Registration number: ChiCTR2200063065, First registered on: 30/08/2022) and the Ethics Committee of the First Hospital of Jilin University (Approval Number: 2019-361). The patient information and blood samples involved in this study have obtained informed consent from all participants and/or their legal guardians. All methods described in this study were conducted in strict accordance with the relevant guidelines and regulations.

### Protocol and registration

Study Protocol Patients were administered an infusion model of meropenem (manufacturer: Hanhui Pharmaceuticals Co., Ltd.; product batch number: 21113311) at a dose of 1.0 g/30 min via intravenous injection within 1 h of admission to the ICU (50 ml of normal saline solution plus 1.0 g of meropenem). Non-drug site blood samples (2 ml) were collected at 0.25 h, 0.5 h, 1.0 h, 1.5 h, 2.5 h, 3.5 h, 5.5 h, and 7.5 h after administration of the first dose of meropenem. After blood collection, the blood was injected into a EDTA anticoagulant tube and then centrifuged at 3000r for 20 min to separate the plasma. The plasma was stored in a − 80 °C freezer until further analysis.

### Plasma sample

Several 1.5 mL Eppendorf (EP) tubes were taken and labeled with unique numbers. Then, 30 μL of the test plasma sample was accurately aspirated into each tube, followed by the addition of 30 μL of internal standard solution (meropenem-d6, concentration 10 μg/mL). Next, 840 μL of acetonitrile was added, and the mixture was vortexed for 5 min. Subsequently, the tubes were centrifuged at a speed of 13,300 rpm for 5 min, and 2 μL of the supernatant was aspirated for analysis using liquid chromatography-tandem mass spectrometry (LC–MS/MS) for qualitative and quantitative analysis^20,21^. Representative chromatograms of meropenem and the internal standard, and the specific experimental scheme is in the Supplementary materials.

### PK analysis

Blood samples were taken from patients at 8 time points between dosing and then plasma samples were tested using the LC–MS/MS method. Subsequently, the obtained concentration–time data were analyzed using non-compartmental modeling in the Phoenix 8.1 pharmacokinetic software. This analysis allowed for the determination of the PK parameters for each patient.

Subgroup analysis was performed according to the patients' Creatinine clearance (CrCl), and PK parameters of CrCl normal subgroup and CrCl abnormal subgroup were obtained respectively.

### PD analysis

Based on the obtained PK parameter information, Monte Carlo simulations were performed using the Crystal Ball software. According to the formula^22,23^, different dosing regimens of 0.5 g/q6h, 1.0 g/q8h, 1.0 g/q6h, and 2.0 g/q12h were simulated under conditions of infusion at 0.5 h, 3 h, and 6 h, against suspected bacterial strains with various MIC values (0.25, 0.5, 1, 2, 4, 8, 16, 32, 64) mg/L. The simulations aimed to determine %fT > MIC (40%, 60%, 80%, 100%) and %fT > 4MIC (40%, 60%, 80%, 100%) for each dosing regimen. A dosing regimen is typically considered reasonable if the PTA is greater than 90%.

### Data collection

General clinical information of the patients was collected, including age, gender, Acute Physiology and Chronic Health Evaluation II (APACHE II) score, Sequential Organ Failure Assessment (SOFA) score, body mass index (BMI), CrCl (Cockcroft-Gault equation estimates^24^), as well as relevant clinical infection indicators such as complete blood count, procalcitonin (PCT), and pathogen identification tests.

### Correlation analysis

Explore the correlation between patients' clinical data and PK parameters.

### Statistical analysis

The statistical software used was SPSS 26.0 (Armonk, NY, USA: IBM Corp). Normally distributed continuous data were expressed as mean ± standard deviation (Mean ± SD). Non-normal distributed continuous data is represented by median (range). Spearman test was used to analyze the correlation between clinical data and PK parameters. Results were considered statistically significant at *P* < 0.05.

### Ethical approval and consent to participate

This study has been approved by the Chinese Clinical Trial Registry (registration number: ChiCTR2200063065); And the approval of the Ethics Committee of the First Hospital of Jilin University (Approval number: 2019-361).

## Results

### Patient characteristics

**Figure 1:**
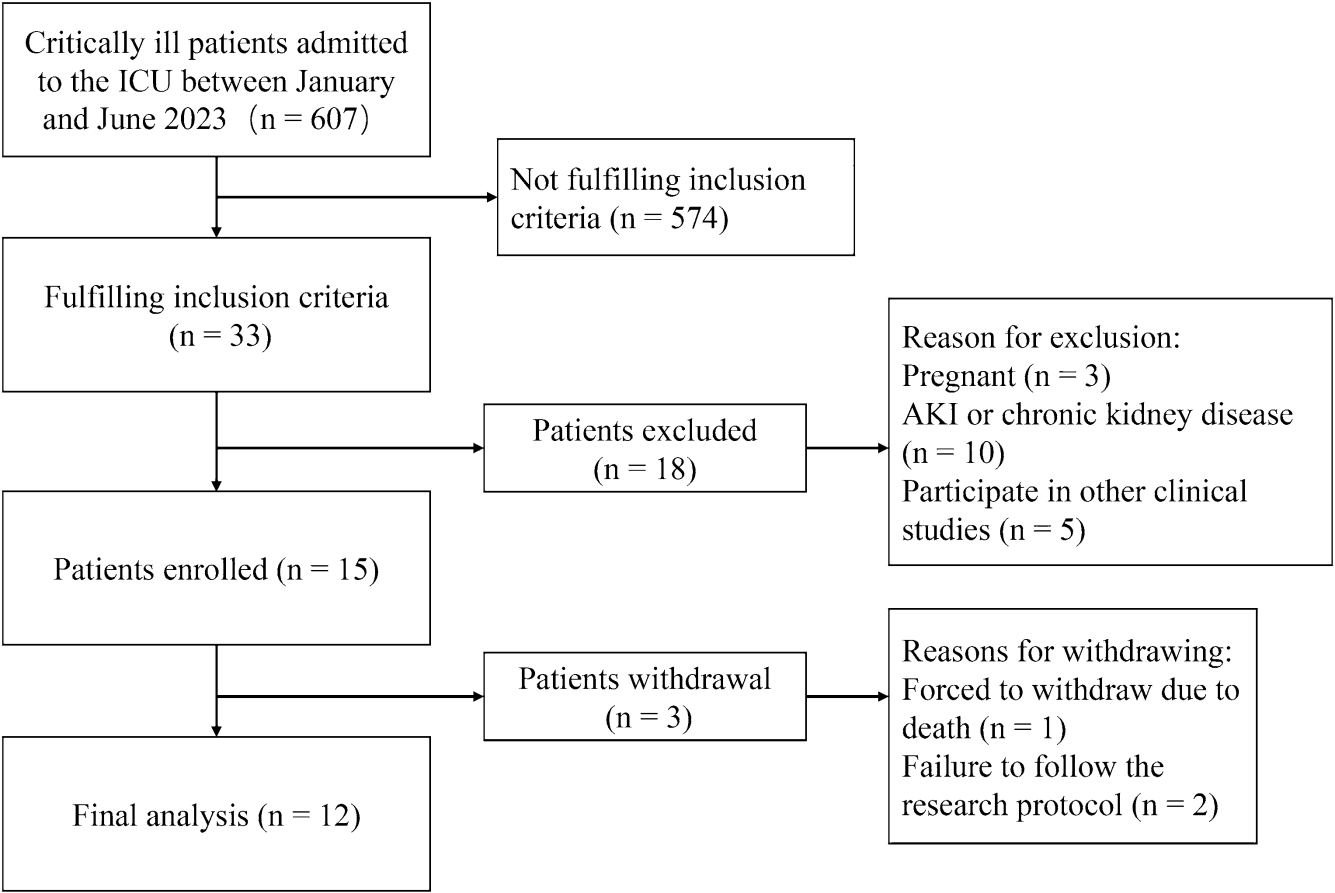
Patient inclusion flowchart.

Out of a total of 607 patients, 33 patients met the inclusion criteria. Three pregnant women, 10 patients with AKI or chronic kidney disease, and five patients participating in other clinical studies were excluded. Of the 15 patients ultimately enrolled, one patient was excluded as he/she died and two patients were excluded due to non-compliance with the blood collection schedule. Therefore, 12 patients (96 blood samples) were ultimately included in this study (Fig. 1), with a mean age of 54.1 ± 17.3 years and a mean BMI of 24.2 ± 3.0. The baseline characteristics of the patients are summarized in Table 1.

**Table 1:** Patient characteristics.

Patient group and number	Age (years)	Gender	BMI (kg/m^2^)	APACHE II score	SOFA score	CrCl (ml/min)	MAP (mmHg)	White blood cell count (× 10^^^9/L)	PCT (ug/L)	Pathogen and MIC (mg/L)
CrCl normal subgroup										
1	23	Male	17.9	11	3	160.9	60	14.8	2.6	*Candida albicans*
2	75	Male	23.6	26	13	54.2	60	20.1	> 100	*Pseudomonas aeruginosa* (MIC = 4 mg/L), *Klebsiella pneumoniae* (MIC ≤ 0.25 mg/L) and *Streptococcus viridis*
3	73	Female	25.4	31	11	91.4	44	14.7	14	*Enterococcus faecium* (MIC = 8) and E. coli (MIC ≤ 0.25)
4	41	Male	25.8	17	5	87.1	54	15.8	3.2	*E. coli* (MIC ≤ 0.25)
5	70	Male	27.1	25	11	55.0	58	18.6	> 100	Klebsiella pneumoniae (MIC = 2)
6	42	Male	21.2	16	4	90.3	42	13.0	0.23	*E. coli* (MIC ≤ 0.25)
7	31	Male	26.2	18	6	168.4	47	6.8	13	NR
8	57	Female	19.8	16	9	125.5	64	14.9	28.7	Klebsiella pneumoniae (MIC ≤ 0.25)
9	49	Female	24.5	24	8	109.1	60	17.4	5.5	NR
CrCl abnormal subgroup										
10	52	Male	26.0	26	11	25.4	62	30.2	37	*E. coli* (MIC ≤ 0.25) and Klebsiella pneumoniae (MIC ≤ 0.25)
11	71	Female	25.6	25	8	47.0	53	13.1	6.7	*E. coli* (MIC ≤ 0.25)
12	65	Female	27.4	17	9	42.7	60	18.9	0.8	*E. coli* (MIC = 2) and Enterobacter cloacae (MIC ≤ 0.25)

CrCl, Creatinine clearance; BMI, body mass index; MAP, mean arterial pressure; PCT, Procalcitonin; APACHE, Acute Physiology, and Chronic Health Evaluation; SOFA, Sequential Organ Failure Assessment; E.coli, escherichia coli; MIC, minimum inhibitory concentration; NR, not reported.

### Population PK analysis

We monitored the blood concentration of meropenem after the initial dose in 12 adult patients with abdominal septic shock caused by gastrointestinal perforation. PK parameters of the 12 patients were calculated using the non-compartmental model implemented in Phoenix 8.1 software. The area under the concentration–time curve (AUC) of 12 patients was 140.5 ± 47.1 mg h/L; the V_d_ was 21.3 ± 5.6 L; the CL was 6.9 ± 2.2 L/h; the maximum concentration (C_max_) was 61.4 ± 9.6 mg/L; and the half-life (T_1/2_) was 2.6 ± 0.5 h.

We further conducted subgroup analyses based on patients' normal CrCl (CrCl > 50 ml/min). In the normal CrCl subgroup, the AUC was 120.9 ± 27.3 mg h/L, the V_d_ was 22.6 ± 5.1 L, the CL was 7.7 ± 1.8 L/h, the C_max_ was 58.7 ± 8.8 mg/L, and the T_1/2_ was 2.6 ± 0.5 h. In the abnormal CrCl subgroup, the AUC was 199.4 ± 47.7 mg h/L, the V_d_ was 17.2 ± 5.8 L, the CL was 4.4 ± 1.1 L/h, the C_max_ was 69.4 ± 7.9 mg/L, and the T_1/2_ was 2.9 ± 0.5 h (Table 2).

**Table 2:** PK parameters of the patient.

Patient group and number	AUC (mg h/L)	CL (L/h)	V_d_ (L)	C_max_ (mg/L)	T_max_ (h)	T_1/2_ (h)
CrCl normal subgroup						
1	81.5	10.6	32.9	43.2	0.25	3.5
2	141.1	6.0	20.9	64.3	0.25	2.7
3	134.3	7.1	15.1	69.8	0.5	1.8
4	137.6	6.4	20.1	53.1	0.5	2.4
5	115.2	7.8	22.4	64.1	0.25	2.4
6	116.3	7.9	21.6	50.7	0.5	2.2
7	97.1	9.6	24.7	54.1	0.25	2.1
8	168.9	4.9	19.0	62.6	0.25	2.9
9	96.3	9.1	27.0	66.8	0.25	3.0
Mean ± SD	120.9 ± 27.3	7.7 ± 1.8	22.6 ± 5.1	58.7 ± 8.8	0.25 (0.25, 0.5)	2.6 ± 0.5
CrCl abnormal subgroup						
10	144.3	5.6	23.9	62.0	0.25	3.3
11	225.0	3.9	13.0	77.8	0.25	2.3
12	228.8	3.6	14.8	68.5	0.5	3.0
Mean ± SD	199.4 ± 47.7	4.4 ± 1.1	17.2 ± 5.8	69.4 ± 7.9	0.25 (0.25, 0.5)	2.9 ± 0.5
Total Mean ± SD	140.5 ± 47.1	6.9 ± 2.2	21.3 ± 5.6	61.4 ± 9.6	0.25 (0.25, 0.5)	2.6 ± 0.5

AUC, area under the concentration–time curve; CL, clearance rate; V_d_, apparent volume of distribution; C_max_, maximum Concentration; T_max_, time to reach maximum concentration; T_1/2_, half-life. Values are Mean ± SD or median (range).

### Monte Carlo simulation

In this study, the PK parameters of septic patients with abdominal infection were obtained. Monte Carlo simulation was performed using Crystal Ball software. The parameter settings for Monte Carlo simulation were as follows: MIC followed a customized distribution with values of 0.25, 0.5, 1, 2, 4, 8, 16, 32, and 64 mg/L. In CrCl normal subgroup, V_d_ was set to (22.6 ± 5.1) L, CL was set to (7.7 ± 1.8) L/h; in CrCl abnormal subgroup, V_d_ was set to (17.2 ± 5.8) L, CL was set to (4.4 ± 1.1) L/h, and both V_d_ and CL followed a normal distribution with a range greater than 0. T_inf_ (h) and Dose (mg) followed a customized distribution according to the chosen infusion protocol for simulation. The value of f ranged from 0.85 to 0.98^23^ and was set to a uniform distribution. The simulation was conducted 10,000 times with a confidence interval of 95%.

#### *PK/PD target is %fT* > *MIC (*Figs. 2 and 3*)*

**Figure 2:**
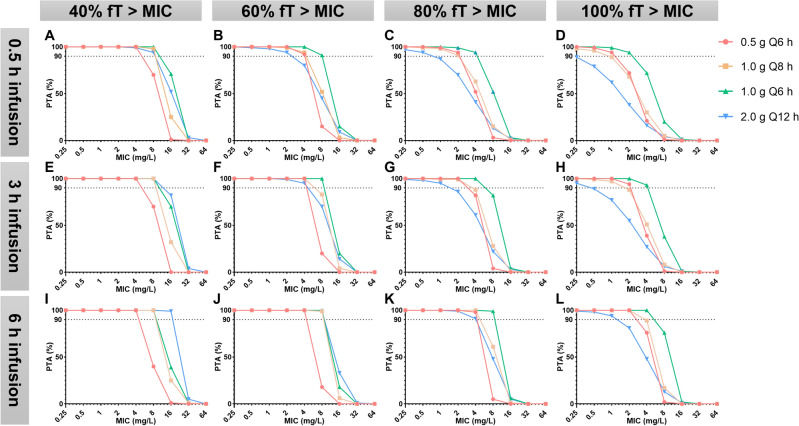
PK/PD target is %fT > MIC (CrCl normal subgroup).

**Figure 3:**
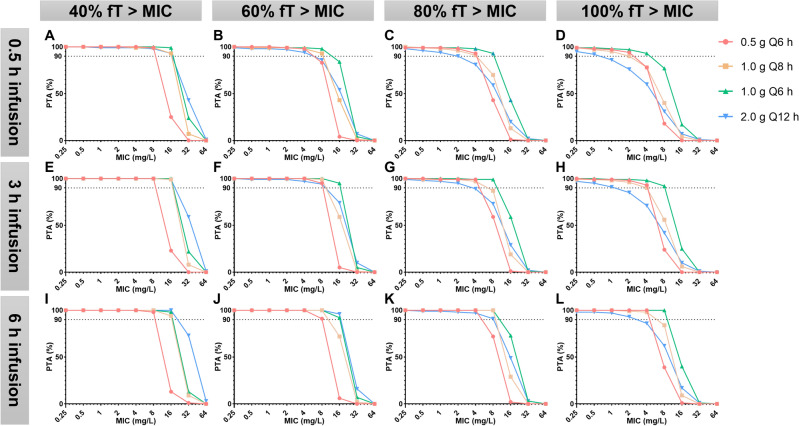
PK/PD target is %fT > MIC (CrCl abnormal subgroup).

##### Target of 40%fT > MIC

No matter in which subgroup, all dosing regimens achieved a PTA > 90% when MIC ≤ 4 mg/L.

In the CrCl normal subgroup, except for the 0.5 g/q6 h regimen, all other regimens met the target PTA when MIC was 8 mg/L.

In the CrCl abnormal subgroup, except for the 0.5 g/q6 h regimen, all other regimens met the target PTA when MIC was 16 mg/L.

##### Target of 60–100%fT > MIC

In the CrCl normal subgroup, the 0.5 g/q6 h regimen infused 0.5 h achieved a PTA > 90% when MIC ≤ 1 mg/L.

In the CrCl abnormal subgroup, the 0.5 g/q6 h regimen infused 0.5 h achieved a PTA > 90% when MIC ≤ 2 mg/L.

Compared to infusion over 0.5 h, almost all regimens with a infusion over 6 h increased the probability of achieving PTA > 90%.

Among the different regimens, the 1.0 g/q6 h regimen infused over 6 h had the highest probability of achieving PTA > 90%.

#### *PK/PD target is %fT* > *4MIC (*Figs. 4 and 5*)*

**Figure 4:**
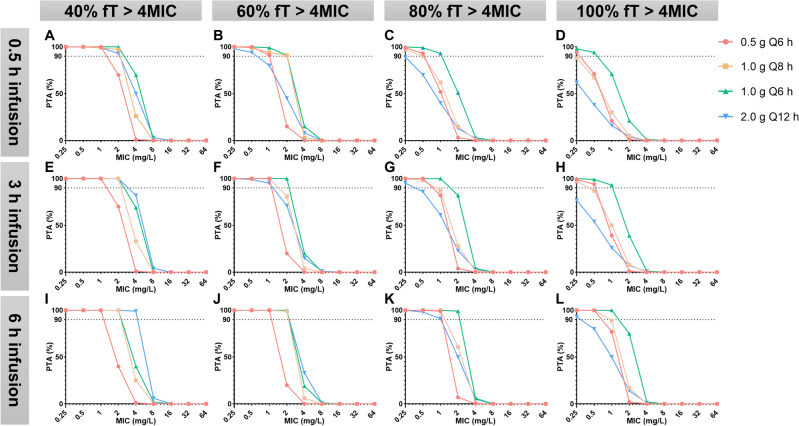
PK/PD target is %fT > 4MIC (CrCl normal subgroup).

**Figure 5:**
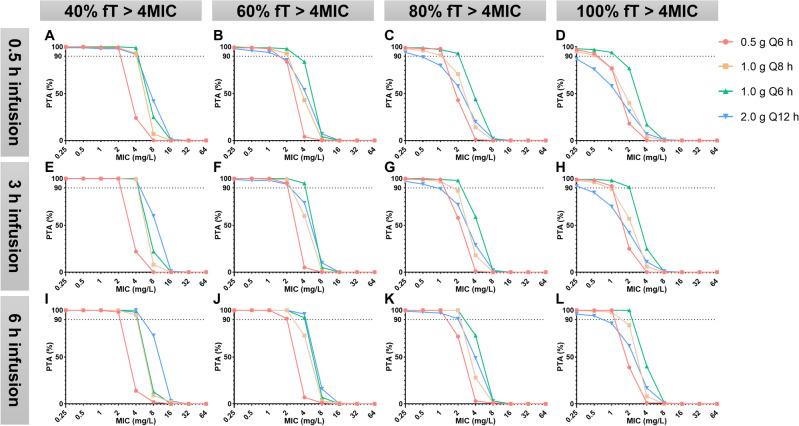
PK/PD target is %fT > 4MIC (CrCl abnormal subgroup).

##### Target of 40%fT > 4MIC

Regardless of the subgroup, all dosing regimens achieved a PTA > 90% when MIC ≤ 0.5 mg/L.

In the CrCl normal subgroup, when MIC = 1 mg/L, all regimens except for the 2.0 g/q12 h infusion over 0.5 h achieved PTA > 90%.

In the CrCl normal subgroup, when targeting 40%fT > 4MIC with MIC = 4 mg/L, only the 2.0 g/q12 h infusion over 6 h regimen achieved PTA > 90%.

##### Target of 60–100%fT > 4MIC

Regardless of the subgroup, except for the 2.0 g/q12 h regimen, all dosing regimens achieved a PTA > 90% when MIC ≤ 0.25 mg/L.

In the CrCl normal subgroup, when targeting 80%fT > 4MIC with MIC = 2 mg/L, only the 1.0 g/q6 h infusion over 6 h regimen achieved PTA > 90%; When targeting 100%fT > 4MIC with MIC = 1 mg/L, only the 1.0 g/q6 h infusion over 3 or 6 h regimen achieved PTA > 90%.

In the CrCl abnormal subgroup, when targeting 100%fT > 4MIC with MIC = 2 mg/L, only the 1.0 g/q6 h infusion over 3 or 6 h regimen achieved PTA > 90%.

### Correlation analysis

Correlation analysis was conducted between clinical data of patients and PK parameters (Cl and V_d_) (Tables 3 and 4). There was negative correlation between age (*P* = 0.036) and Cl, and positive correlation between CrCl (*P* = 0.007) and Cl. Age (*P* = 0.024) was negatively correlated with V_d_.

**Table 3:** Correlation analysis of patient clinical data and clearance.

Clinical data	Correlation coefficient	*P* value
Age	− 0.608	0.036*
Gender	− 0.241	0.450
BMI	− 0.294	0.350
APACHE II score	− 0.239	0.454
SOFA score	− 0.488	0.108
CrCl	0.727	0.007**
MAP	− 0.335	0.288
White blood cell	0.413	0.183
PCT	− 0.172	0.594

CrCl, Creatinine clearance; BMI, body mass index; MAP, mean arterial pressure; PCT, Procalcitonin; APACHE, Acute Physiology, and Chronic Health Evaluation; SOFA, Sequential Organ Failure Assessment; *P < 0.05; **P < 0.01.

**Table 4:** Correlation analysis of patient clinical data and volume of distribution.

Clinical data	Correlation coefficient	*P* value
Age	− 0.643	0.024*
Gender	− 0.386	0.215
BMI	− 0.203	0.527
APACHE II score	− 0.204	0.524
SOFA score	− 0.304	0.337
CrCl	0.469	0.124
MAP	0.167	0.603
White blood cell	0.035	0.914
PCT	0.014	0.966

CrCl, Creatinine clearance; BMI, body mass index; MAP, mean arterial pressure; PCT, Procalcitonin; APACHE, Acute Physiology, and Chronic Health Evaluation; SOFA, Sequential Organ Failure Assessment; *P < 0.05; **P < 0.01.

## Discussion

Severe sepsis and septic shock are among the leading causes of death in ICU patients^25^. A multi-center study worldwide showed that the abdominal cavity is the most common source of sepsis, second only to the respiratory tract^26^. Moreover, abdominal sepsis caused by gastrointestinal perforation has a worse prognosis than sepsis from other sources^4^. Therefore, exploring the PK parameters of meropenem in patients with abdominal sepsis is of great significance for the treatment and prognosis of critically ill patients. In this study, monitoring of blood drug concentrations was performed at various time points (0.25 h, 0.5 h, 1.0 h, 1.5 h, 2.5 h, 3.5 h, 5.5 h, 7.5 h) after the initial dose of meropenem in 12 patients with abdominal septic shock caused by digestive tract perforation in the ICU. In the normal and abnormal CrCl subgroups, the following PK parameters were obtained: V_d_ was 7.7 ± 1.8 and 4.4 ± 1.1, respectively. Cl was 22.6 ± 5.1 and 17.2 ± 5.8, respectively. There were also some differences in other PK parameters between subgroups. Overall, V_d_ was slightly higher in the study patient population compared to PK parameters in healthy volunteers. This may be related to fluid resuscitation during shock, the use of positive inotropic drugs, fluid extravasation due to capillary leakage and tissue edema.CL was significantly lower than that in healthy volunteers, which may be attributed to decreased renal perfusion caused by shock; C_max_ in normal and abnormal CrCl subgroups was 58.7 ± 8.8 and 69.4 ± 7.9 mg/L, respectively, which was comparable to that in healthy volunteers (55.4–61.6 mg/L); T_1/2_ in normal and abnormal CrCl subgroups was 2.6 ± 0.5 and 2.9 ± 0.5 h, significantly prolonged compared to healthy volunteers (0.96–0.98 h); the AUC in normal and abnormal CrCl subgroups was 120.9 ± 27.3 and 199.4 ± 47.7 mg h/L, increased compared to healthy volunteers (66.9–77.5 mg h/L), possibly due to decreased drug clearance and prolonged half-life leading to drug accumulation^23,27^. It is evident that there are significant changes in the PK parameters of patients with abdominal sepsis compared to healthy individuals^23^. Although our study focused on critically ill patients with abdominal septic shock, the differences in V_d_ (23.7 L) and CL (7.8 L/h) compared to the meropenem PK parameters established by Sutep et al.^28^ in a population model of severe sepsis are relatively small. Moreover, compared with the meropenem PK parameters in the sepsis population model established by Goncalves-Pereira et al.^29^, Vd (18.5–17.3 L) and CL (7.2–8.1 L/h) had relatively small differences. This study also found that age was negatively correlated with Vd and Cl, while CrCl was positively correlated with Cl.

The latest sepsis guidelines recommend prolonging the infusion time of β-lactam antibiotics to achieve better therapeutic efficacy^19^. Furthermore, meta-analysis has shown that extending the infusion time of β-lactam antibiotics can reduce the short-term mortality rate in critically ill septic patients^30,31^. Based on the obtained PK parameters of meropenem in patients with abdominal sepsis, this study provides a basis for the rational application of meropenem in clinical practice through simulating different dosing regimens. The results of this study indicate that when the treatment target is set at 40% fT > MIC, an administration regimen of 0.5 g/q6 h infused over 0.5 h can achieve a PTA > 90% for MIC ≤ 4 mg/L. Even when the most optimal bactericidal target of 100% fT > 4MIC is considered as the treatment goal, the administration regimen of 0.5 g/q6 h infused over 0.5 h can still achieve a PTA > 90% for MIC ≤ 0.25 mg/L. However, when 100%fT > 4MIC was the target and MIC = 0.5, 0.5 g/q6 h infusion over 0.5 h regimen could not make PTA > 90%, while 0.5 g/q6h infusion over 6 h regimen could make PTA > 90%. Only the infusion time was extended, thus achieving better antimicrobial efficacy. Therefore, the infusion regimen of 0.5 g/q6h infusion over 6 h may be more suitable for patients with abdominal sepsis with a lower MIC. It is important to individualize antibiotic therapy for critically ill patients and not blindly follow traditional dosing regimens based on the actual condition of the patient. A study conducted in the United States reported significantly high hospitalization costs for septic patients, labeling sepsis as one of the costliest diseases in a hospital environment^32^. In Chinese hospitals, with the popularization of diagnosis related groups (DRGs) as a payment system, reducing antibiotic usage and medical expenses, as well as lowering the defined daily dose (DDD) of antibiotics, should gradually become a treatment goal^33^. Therefore, for patients with abdominal sepsis who are at low MIC, the administration regimen of 0.5 g/q6h infused over 6 h not only achieves higher %PTA but also reduces the dose of meropenem, thereby achieving optimal cost-effectiveness. For patients with low MIC infection, the effectiveness and clinical value of this regimen need to be confirmed by further studies.

However, for critically ill patients with abdominal septic shock with high MIC, maximizing the effectiveness of antimicrobial therapy is particularly important. This study demonstrates that the administration regimen of 1.0 g/q6 h infused over 6 h has the highest probability of achieving a PTA > 90% and shows superior antimicrobial efficacy. In a study by Sutep et al.^28^. with similar PK parameters to this study, an administration regimen of 2.0 g/q8 h infused over 4 h only achieved a PTA > 90% when the MIC ≤ 2 mg/L, whereas in this study, an administration regimen of 1.0 g/q6 h infused over 6 h achieved a PTA > 90% when the MIC was 8 mg/L. These results suggest that extending the infusion time of meropenem and shortening the dosing interval can significantly reduce the drug dosage while achieving better PK/PD effects. Therefore, for patients with abdominal sepsis with high MIC, a dose regimen of 1.0 g/q6 h infusion over 6 h can achieve higher %PTA and lower metabolic burden on organs (smaller daily drug dose) compared to a dose regimen of 2.0 g/q8 h infusion over 4 h. The antibacterial effect and clinical value of this administration regimen still need to be verified by high-quality studies.

Although we performed a subgroup analysis based on whether the patients' CrCl was normal, the results suggest some differences in PK parameters between subgroups. However, from the results of the Monte Carlo simulation, it appears that the administration regimen of 0.5 g/q6 h infusion over 6 h for infections with low MIC and 1.0 g/q6h infusion over 6 h for infections with high MIC, respectively, is equally appropriate for patients with normal or abnormal CrCl. The results of this study suggest that Vd and Cl are negatively correlated with age, while Cl is positively correlated with CrCl. Due to the small sample size of the study and the large uncertainty of the results, we can only interpret this result cautiously, and large-scale experiments are still needed to verify this conclusion.

This study has several limitations. Firstly, although all 12 patients included in the study had postoperative abdominal sepsis after gastrointestinal perforation, there were still significant differences in PK parameters among individuals. Even though we performed subgroup analyses to reduce the impact of large differences in CrCl among patients, a small sample size was insufficient to draw reliable conclusions, and further intervention studies with larger sample sizes are needed to accurately determine the PK parameters of abdominal sepsis patients. Secondly, due to the small sample size and the presence of various types of pathogens, including cases of polymicrobial infections, we did not compare the microbiological results among patient groups. Thirdly, the model established in this study can only predict the PK parameters of early-stage meropenem in abdominal sepsis. Therefore, its value in guiding the initial dosing regimen of meropenem for sepsis in the middle and late stages is unknown. Finally, regarding the PK parameters of meropenem for complex intra-abdominal infections caused by multidrug-resistant bacteria, further research and verification are still needed based on the microbiological results.

## Conclusions

Patients with septic shock after severe gastrointestinal perforation show altered pharmacokinetic parameters of meropenem, characterized by increased apparent volume of distribution, decreased clearance, and prolonged half-life. For infections with low MIC, a dosing regimen of 0.5 g every 6 h infused over 6 h may be preferable. However, for infections with high MIC, a dosing regimen of 1.0 g every 6 h infused over 6 h may be preferable. Further research is needed to confirm this exploratory result. Clinical monitoring of meropenem blood concentrations is necessary to optimize the dosing regimen using PK/PD principles.

### Supplementary Information

Supplementary Information.

## Data Availability

All data generated or analyzed during this study are included in this published article.

## Abbreviations

ICU: Intensive care unit
PTA: Probability of target attainment
MIC: Minimum inhibitory concentration
CrCl: Creatinine clearance
CL: Clearance
V_d_: Volume of distribution
T_1/2_: Half-life
LC–MS/MS: Liquid chromatography-tandem mass spectrometry
AUC: Area under the concentration–time curve
C_max_: Maximum concentration
APACHE II: Acute Physiology and Chronic Health Evaluation II
SOFA: Sequential Organ Failure Assessment
BMI: Body mass index
PCT: Procalcitonin
DRGs: Diagnosis related groups
DDD: Defined daily dose
