# Supplementary material for: Changes of PK/PD of Meropenem in patients with abdominal septic shock and exploration of clinical rational administration plan: a prospective exploratory study
Source: Sci Rep. 2024 May 3;14:10173. doi: 10.1038/s41598-024-60909-7 (PMC11068909; doi:10.1038/s41598-024-60909-7)
Supplement: Supplementary file 1 — Supplementary Information. [file 41598_2024_60909_MOESM1_ESM.docx]

**Supplementary materials**

**1.** Measurement of meropenem blood concentration using high-performance liquid chromatography-tandem mass spectrometry (HPLC-MS/MS)

**1.1.** Experimental instrument

Instruments and equipment used in this study included an AB5500 QTRAP triple quadrupole mass spectrometer equipped with an electrospray ionization (ESI) source and Analyst1.5 data processing software (AB Sciex, USA), an LC-20A HPLC system (Shimadzu, Japan), a Vortex-Genius 2 vortex mixer (Scientific Industries, USA), a PICO 17 centrifuge (Thermo Fisher Scientific, USA), a KQ-1000KDB ultrasonic cleaner (Kunshan Ultrasonic Instruments Co., Ltd.), a MilliQ Direct-Q16 water purification system (Merck Millipore, USA), and a DENVER TB215D analytical balance (Denver Instrument, USA).

**1.2.** Experimental drug

The drugs used in this study were meropenem (Sigma-Aldrich, USA; purity ≥97%), meropenem-d6 (Spectrum Chemical Mfg. Corp., China; purity ≥99.5%), formic acid (Sigma-Aldrich, USA), acetonitrile (Sigma-Aldrich, USA), and MilliQ ultra-pure water. **1.3.** Chromatographic conditions and columns

Chromatographic separation was achieved using an Agilent Poroshell120 SB-C18 column (4.6 mm × 50 mm, 2.7 μm) with a column temperature of 40°C. The injection volume was 2.0 μL, and the mobile phase consisted of 0.1% formic acid aqueous solution (A) and acetonitrile (B). The composition and flow rate of the mobile phase gradient are shown in Table 1.

**Table S1.** Mobile phase gradient composition and flow rate

| Time (min) | Flow rate (mL·min^-1^) | A (0.1% formic acid) | B (Acetonitrile) |
| --- | --- | --- | --- |
| 0.01 | 0.7 | 95 | 5.0 |
| 0.50 | 0.7 | 95 | 5.0 |
| 2.50 | 0.7 | 5.0 | 95 |
| 4.50 | 0.7 | 5.0 | 95 |
| 4.6 | 0.7 | 95 | 5 |
| 7.0 | 0.7 | 95 | 5 |

**1.4.** Mass spectrum condition

The mass spectrometry analysis was performed using an electrospray ionization source (ESI) with high-purity nitrogen gas (>99.99%) as the nebulizing and curtain gas. The scan mode was set to positive ionization, and the detection method was multiple reaction monitoring (MRM). The ESI voltage was set to 5500 V, and the ion source temperature was maintained at 500 ℃. The nebulizing gas pressure (Gas1) and curtain gas pressure (Gas2) were both set to 50 psi, while the collision gas pressure (CUR) was set to 30 psi with moderate collision energy. The entrance potential (EP) was set to 10 V, and the collision cell exit potential (CXP) was set to 12.0 V. The MRM monitored ion pairs, declustering potential (DP), and collision energy (CE) are detailed in Table 2.

**Table S2.** Mass spectrum parameters of meropenem and its isotope internal targets

| Drug | Precursor ion (m/z) | Product ion (m/z) | Declustering voltage (V) | Collision energy (eV) |
| --- | --- | --- | --- | --- |
| Meropenem | 384.0 | 141.0 | 80 | 18 |
| Meropenem -d6 | 390.0 | 147.0 | 80 | 20 |

**1.5.** Preparation of standard solution

reparation of standard and quality control solutions: Accurately weigh 10 mg of meropenem reference standard in a 10 mL volumetric flask. Dissolve it in acetonitrile-water (50/50, v/v) and dilute to the mark, shake well, and prepare a 1 mg/mL meropenem stock solution. Take appropriate amounts of the stock solution and dilute with blank plasma to obtain standard series solutions with meropenem plasma concentrations of 0.5, 1, 2, 5, 10, 20, 30, and 50 μg/mL respectively, as required for the standard curve. Take suitable amounts of the stock solution and dilute with blank plasma to prepare low, medium, and high quality control solutions with meropenem plasma concentrations of 1.5, 8, and 40 μg/mL, respectively, as required for the quality control samples. Preparation of internal standard solution: Accurately weigh 1 mg of meropenem-d6 reference standard, add 1 mL of acetonitrile-water (50/50, v/v), dissolve and mix well, and prepare a 1 mg/mL meropenem-d6 stock solution. Store it in a refrigerator at -20°C for later use. Dilute the internal standard stock solution to a concentration of 10 μg/mL to obtain the internal standard solution, which should be stored in a refrigerator at -20°C for later use.

**1.6.** Plasma samples

Take several 1.5 mL EP tubes and label them accordingly. Precisely pipette 30 μl of the test plasma sample into each tube. Add 30 μl of the internal standard solution (meropenem-d6, 10 μg/mL) and then add 840 μl of acetonitrile. Mix the contents by vortexing for 5 minutes, then centrifuge at a speed of 13,300 rpm for 5 minutes. Take 2 μl of the supernatant for LC-MS/MS analysis. Representative chromatograms of meropenem and the internal standard are shown in Figure S1.


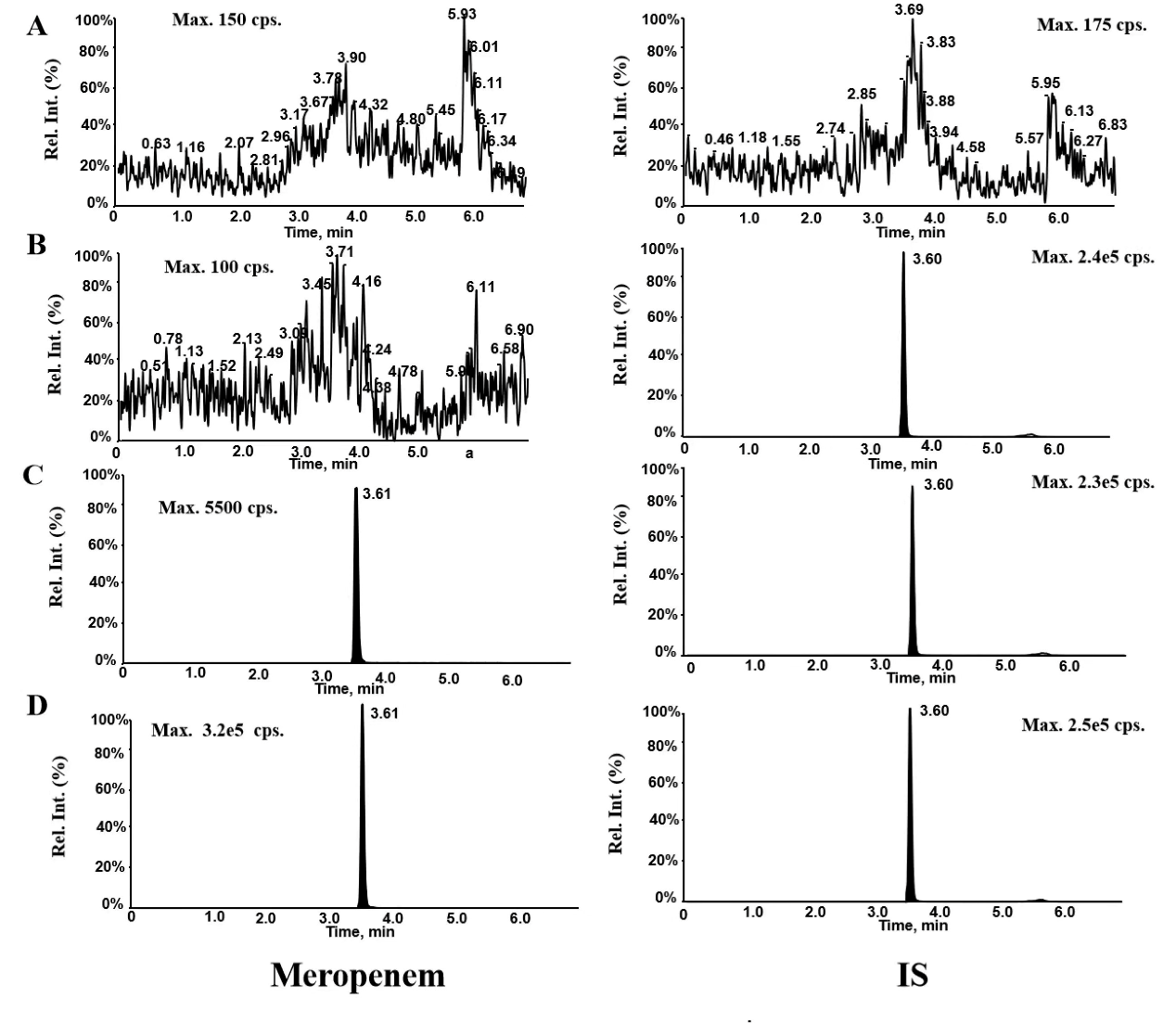


**Figure S1.** Representative chromatographic diagram of meropenem and internal standard in human plasma. (A) Blank plasma samples; (B) Blank plasma with internal standard sample (10μg/mL); (C) Blank plasma plus control (0.5μg/mL) and internal standard (10µg/mL) samples; (D) Actual human plasma samples.
